# The effects of birth weight and gender on neonatal mortality in north central Nigeria

**DOI:** 10.1186/1756-0500-4-562

**Published:** 2011-12-24

**Authors:** Caroline A Onwuanaku, Seline N Okolo, Kemi O Ige, Sylvanus E Okpe, Bose O Toma

**Affiliations:** 1 Department of Paediatrics, Jos University Teaching Hospital, Jos, Nigeria

**Keywords:** Birth weight, Gender, Gestational age, Neonatal mortality, North central, Nigeria

## Abstract

**Background:**

Worldwide 15.5% of neonates are born with low birth weight, 95.6% of them in the developing countries. Prematurity accounts for 10% of neonatal mortality globally. The purpose of this study was to evaluate the effects of birth weight and gender on neonatal outcome.

**Findings:**

The data of 278 neonates managed in the Special Care Baby Unit (SCBU) of Jos University Teaching Hospital (JUTH) over a 2 year period from July 2006 to June 2008 were analyzed.

One hundred and fifty nine (57.2%) were males and 119(42.8%) females. There were 87(31.3%) preterm and 191 (68.7%) term babies. Twelve of the babies died. Seven (2.52%) and 5 (1.80%) being males and females respectively. The neonatal mortality rate by gender was not significant (p > 0.05). The neonatal mortality was 25.2 deaths per 1000 live births for boys and 18.0 for girls. The mean birth weights of the preterm and term babies were 1.88 ± 0.47 kg and 3.02 ± 0.50 kg respectively, with a mean gestational age of 30.62 ± 3.65 weeks and 38.29 ± 0.99 weeks respectively.

Eighty seven (31.3%) of the babies were of low birth weight, 188(67.6%) were of normal birth weight and 3(1.1%) high birth weight. Of the low birth weight babies, 6(2.2%) were term small for gestational age. Six (2.2%) of the preterm infants had normal birth weight.

Eleven of the babies that died were preterm low birth weight. The overall mortality rate was 4.32%. The birth weight specific mortality rate was 126 per 1000 for the preterm low birth weight and 5 per 1000 for the term babies. Birth weight unlike gender is a significant predictor of mortality, mortality being higher in neonates of <2.5 kg (OR = 0.04; 95% Cl 0.005-0.310, p = 0.002) (p = 0.453). Seven (58.3%) and 4(33.3%) of the pre-terms that died were appropriate and large for gestational age respectively. Gestational age is not a significant predictor of neonatal mortality (p = 0.595). Babies delivered at less than 37 weeks of gestation recorded a higher rate of mortality than those of 37 weeks and above (p = 0.000).

The subjects showed one or more major clinical indications for admission. The major clinical indications for the preterm and term babies were respectively as follows: neonatal sepsis 63(22.7%) and 124(44.6%); neonatal jaundice 32(11.1%) and 71(24.7%); malaria 9(3.1%) and 13(4.5%); birth asphyxia 3(1.0%) and 7(2.4%). Neonatal sepsis was a common denominator among the babies that died.

**Conclusion:**

Birth weight unlike gender is a significant predictor of neonatal outcome

## Literature review

Worldwide, 15.5% of all infants are born with low birth weight, 95.6% of them in the developing countries [1]. Birth weight may be a predictor for survival as low birth weight infants have a greater risk of morbidity and mortality [2,3]. Prematurity an indicator of neonatal immaturity accounts for 10% of neonatal mortality globally [4].

Gender -specific infant mortality varies across nations [5]. Male neonates have been consistently reported to have a higher neonatal mortality rate than their female counterparts [6,7]. This study evaluated the effect of birth weight and gender on neonatal mortality in the Special Care Baby Unit of Jos University Teaching Hospital over a 2 year period.

## Findings

### Research hypothesis

It is hypothesised that birth weight and gender have significant positive effects on neonatal mortality (p < 0.05).

## Methods

This is a retrospective descriptive study. The data of 278 neonates who were managed at the Special Care Baby Unit of Jos University Teaching Hospital, Jos, North- central Nigeria from 1st July 2006 to 30th June 2008 whose specimens were sent to the unit's research laboratory for various routine investigations were analyzed.

Jos is the capital city of Plateau state located between latitude 80°24 'N and longitude 80°32' and 100°38'East [8]. Jos University Teaching Hospital which is the teaching hospital of University of Jos, receives referral from different parts of the state and its neighbouring states. The special care baby unit has a bed capacity of twenty-five (25).

Consent was sought and obtained from the head of the records department of the hospital before extracting data from the patients' hospital records. Ethical clearance or consent of the neonates' parents/care giver was not obtained because the study was undertaken retrospectively. Information extracted from the neonates' records include: sex, gestational age at delivery, birth weight, morbidity, outcome and duration of illness before mortality.

Data were analyzed using Epi-info version 3.3.2 [9]. The mean, standard deviation, percentage, frequency, multiple logistic regressions and Z- test were derived using the Epi-info version 3.3.2 [9].

## Results

Out of 278 neonates studied, 159 (57.2%) were males and 119(42.8%) females. Eighty- seven (31.3%) were preterm and 191(68.7%) term babies (Table 1). Table 1 also shows the mortality rate by gender. Twelve of the babies died. Seven (2.52%) and 5(1.80%), of them being males and females respectively. The neonatal mortality rate by gender was not significant (p > 0.05). The neonatal mortality was 25.2 deaths per 1000 live births for boys and 18.0 for girls.

**Table 1:** Maturity and mortality rate of the babies by gender

	Preterm (%)	Term (%)	Mortality (%)
**GENDER**			

Male	46(16.6)	113(40.6)	7(58.3)

Female	41(14.7)	78(28.1)	5(41.7)

**Total**	87(31.3)	191 (68.7)	12(100)

Table 2 shows the mean birth weight of the preterm and term babies as 1.88 ± 0.47 kg and 3.02 ± 0.50 kg respectively, with a mean gestational age of 30.62 ± 3.65 weeks and 38.29 ± 0.99 weeks respectively. Eighty-seven (31.3%) of the babies were of low birth weight (LBW), 188(67.6%) were of normal birth weight and 3(1.1%) high birth weight. Of the LBW babies, 6(2.2%) were term, small for gestational age (SGA); 81(29.1%) were preterm. Six (2.2%) of the preterm babies had normal birth weight.

**Table 2:** Mean Birth Weight (BW) and gestational age of the preterm and term babies

	Birth weight (kg)	Gestational age (weeks)	**No**.	%
**LOW BW**	**1.84 ± 0.17**	**26.46 ± 1.98**	**87**	**31.3**

Preterm	**1.88 ± 0.47**	**30.62 ± 3.65**	**81**	**29.1**

Term SGA	**2.27 ± 0.02**	**37.33 ± 0.27**	**6**	**2.2**

**NORMAL BW**	**2.60 ± 0.26**	**39.89 ± 1.02**	**188**	**67.6**

Preterm	**2.64 ± 0.04**	**36.00 ± 0.00**	**6**	**2.2**

Term	**3.02 ± 0.50**	**38.29 ± 0.99**	**182**	**65.5**

**Term LGA**	**4.81 ± 1.10**	**38.00 ± 0.00**	**3**	**1.1**

Eleven of the babies that died were preterm. The overall mortality rate was 4.32% (OR = 0.04, 95%Cl 0.005-0.310, p = 0.002) this being higher in babies with birth weight of <2.5 kg (Table 3). The birth weight specific mortality rate was 126 per 1000 for the preterm low birth weight and 5 per 1000 for the term babies.

**Table 3:** Total number admitted, gender and mortality rate by birth weight categories

Birth Weight(kg)Categories	Total Number Admitted	Gender		Mortality	
		**M:**	**F**	**No.:**	**%**

0.5-0.9	2	-	2	-	-

1.0-1.4	13	8	5	2	16.7

105-1.9	30	13	17	7	58.3

2.0-2.4	42	25	17	2	16.7

2.5-2.9	105	60	45	1	8.3

3.0-3.4	45	30	15	-	-

3.5-3.9	35	19	16	-	-

4.0-4.4	5	4	1	-	-

4.5-4.9	0	-	-	-	-

5.0-5.4	0	-	-	-	-

5.5-5.9	0	-	-	-	-

6.0-6.4	1	1	-	-	-

Total	278	159	119	12	100

Two (0.7%) of the pre-terms were delivered at 17-20 weeks of gestational age category and were large for gestational age (LGA). Two (0.7%), 38 (13.7%) and 47 (16.9%) of the pre-terms were small for gestational age (SGA), appropriate for gestational age (AGA) and LGA respectively. 7(58.3%) and 4(33.3%) of the pre-terms that died were AGA and LGA respectively (Table 4).

**Table 4:** Gestational age categories and mortality rate by neonatal maturity

Gestational Age Categories	**No**.	Pre-terms			Terms			Post-terms
		SGA:AGA: LGA			SGA: AGA: LGA			
17-20	2	-	-	2	-	-	-	-
21-24	-	-	-	-	-	-	-	-
25-28	-	-	-	-	-	-	-	-
29-32	26	2	9	15	-	-	-	-
33-36	23	-	22	1	-	-	-	-
37-40	187	-	-	-	6	17 8	3	-
41-44	4	-	-	-	-	-	-	4
	
Mortality	12	-	7	4	-	1	-	-

Table 5 shows the multiple logistic regression of mortality with birth weight, gender and gestational age. Birth weight unlike gender is a significant predictor of neonatal mortality p = 0.002 and p = 0.453 respectively. Though the effect of gestational age is positive on mortality, this was not significant. Gestational age is not a significant predictor of neonatal mortality (p = 0.595).

**Table 5:** Multiple logistic regression model of mortality with birth weight (BW), gender and gestational age (GA)

				Number of observations = 278 LR chi2 (2)= 18.72 Prob>chi2 = 0.0001 Pseudo R2 = 0.2025		
Log likelihood=-36.866136						

Mortality	Odds	Std.Error	Z	p >/z/	95%cf	Interval

	ratio					

BW	0.039	0.041	3.07	0.002	0.005	0.310

Gender	0.609	0.403	0.75	0.453	0.166	2.224

GA	0.943	0.110	0.53	0.595	0.274	0.157

Additional file 1: Table S1 showed that there is a significant difference in the mortality rate of babies with gestational age lower than 37 weeks and those of or greater than 37 weeks gestational age (p = 0.000). Neonatal mortality tends to occur more at gestational age lower than 37 weeks.

Additional file 1: Table S2 shows that, neonatal sepsis (NNS) was a common denominator among the babies that died. 8(66.7%) of the preterms that died had NNS.

**Figure 1:**
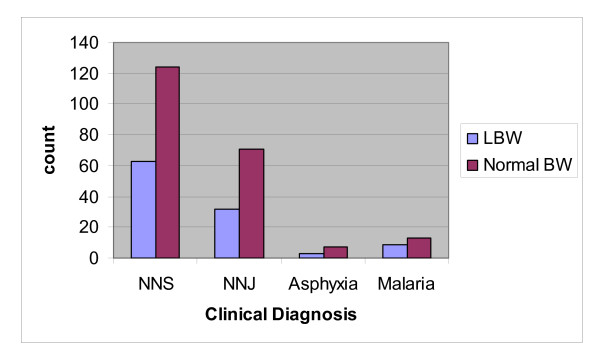
**Major Clinical Indications for Admission by Birth Weight**.

The major clinical indications for admission were shown in Figure 1.The babies had one or more major clinical indications for admission. Neonatal sepsis was the most common indication seen in 63 (22.7%) of the LBW babies and 124 (44.6%) of the babies with normal birth weight. The least common indication was birth asphyxia. This was seen in 10(3.5%) of the babies, 3(1.0%) occurring in the LBW babies. Other major indications include neonatal jaundice (NNJ) and malaria.

## Discussion

From our study, birth weight is a significant predictor of neonatal mortality. This had been reported by some other workers [2,3]. Though most of the babies in our study were term babies with normal birth weight, the fairly high proportion of them who were preterm with low birth weight recorded the highest mortality rate irrespective of their gender. This may be because the preterms due the immaturity of their organs have more difficulty in adaptation to extra- uterine life [2].

Also, the birth weight specific mortality rate from our study implies that on the average more than one in every ten preterm low birth weight neonates admitted in our unit do not leave the unit alive. This is in consistency with the report of Uthman 2008 [2] that more than one in every ten preterm babies in Nigeria do not survive to their first birthday. However, this is higher than that reported by Clarke et al. 1994, [10] in the UK. This implies that in order to give hope to these innocent beings, it is necessary to improve on the measures to encourage the survival of low birth weight babies in our environment [11].

However, bearing in mind that Nigeria as a developing country, may be lacking in provision of adequate facilities and manpower for care and proper management of these babies in all levels of our health care delivery [12], it may be important that obstetric measures to reduce preterm deliveries as suggested by Lams et al. 2008,[13], be sought and encouraged. This is underscored by the fact that preterm birth from our study is found to be the major cause of low birth weight and this tallies with the report from http://wikipedia.org[2].

More boys than girls in our study died, though this was not significant. This is in consonance with the finding by some authors that male neonates have higher mortality rate than their female counterparts [5-7]. This disproportionality in mortality ratio by gender may be due to certain factors such as genetic factors though this was not investigated in this study. Kana et al., 2006 [5] had earlier attributed atypical male to female infant mortality ratios to genetic factors as well as to social and behavioural attitudes of some adults in favour of boys over girls. The gender- specific mortality rate implies that about 3 boys and 2 girls out of every 100 live births admitted in the unit from 2006-2008 did not leave the unit alive. This is much higher than that reported in Canada in 2007 which was 0.55 for boys and 0.47 for girls. (http://www.hrsdc.gc.ca) [14].

Also from the study, though gestational age is not a good predictor of neonatal mortality, the latter tends to occur more at gestational age less than 37 weeks. This agrees with the finding by Herman and Hastie, 1990 [15]. This implies that measures to encourage an improvement in birth weight should be encouraged without neglecting measures to discourage delivery before 37 weeks of gestation. Wilcox and Skjaerven 1992 [16] reported that gestational age is a strong predictor of perinatal mortality which was contrary to our findings. This contradiction may be due to the fact that we looked at the effect of gestational age alone on neonatal mortality unlike the cited authors who looked at the effect of the two variables together.

Neonatal infection may be a predisposing factor in the mortality of these low birth weight preterm neonates as most of the babies and particularly those that died were clinically diagnosed of neonatal sepsis. This is in agreement with works done in Ebonyi State [11] and Lagos University Teaching Hospitals [12]. This high rate of infections among the preterms is not surprising as this may be due to the immaturity of their organs including the immune system [17]. Thus, low birth weight coupled with neonatal infections may have significantly contributed to the death of the babies in our study. Though, the causative organisms and the routes of the infections were not investigated in our study, it is suggested that, aseptic measures to encourage and improve on the hygienic status of the: unit, mothers/care givers, facilities, equipment and personnel be taken with greater priority.

Finally, if the Millennium Development Goals (MDGs) which is a set of UN priorities slated to be met by 2015, with the fourth goal being on the reduction of the incidence of low birth weight deliveries for reducing child mortality by two thirds [18] is to be a reality, then, our policy makers should in their plans intensify effort in health budgeting and plan execution with primary attention on reducing the incidence of preterm low birth weight and its consequent mortality with particular attention on improving neonatal care service delivery [3,11,12].

## Conclusion

Birth weight unlike gender is a significant predictor of neonatal outcome.

This implies that measures to reduce the rate of preterm low birth weight deliveries and improve on the survival of the preterm low birth weight neonates including intensive asepsis measures should be encouraged and given greater priority.

### Recommendations

Though, some infection control measures like separating inborn and out born, proper dressing of personnel, hand washing practices are in place in the unit there is still a high prevalent rate of sepsis among the babies admitted in the unit, thus it is being recommended that more stringent asepsis measures be put in place in order to reduce the rate of infections and the consequent mortality.

Secondly, we recommend improvement in obstetric care to reduce low birth weight/prematurity rate; and improved neonatal care of low birth weight babies to enhance their survival.

### Limitation of the study

One major limitation of the study was that the data were extracted from the hospital records of babies whose specimens were sent to the unit's research laboratory within the stated time frame for various routine investigations, not necessarily the total number of admissions to the unit. Thus, a further study in which the total number of admissions to the unit is made use of may be necessary.

## Competing interests

The authors declare that they have no competing interests.

## Authors' contributions

OC conceived the idea, participated in data extraction, design, analysis and interpretation of data and drafting of the manuscript; OS participated in the design, interpretation of data and reviewing of the manuscript; IO participated in the design, analysis and interpretation of data and drafting of the manuscript; OE participated in reviewing the paper while TB participated in the design of the work. All the authors went through the final copy of the work and gave their final approval.

## Supplementary Material

Additional file 1 **Table S1. Z-test of Gestational Age (GA) and Mortality, Table S2. Neonatal Mortality by Cause of Death**. Click here for file

## References

[B1] United Nations Children's Fund World Health Organization Low birth weight: country, regional and global estimates 2004 New York: UNICEF 2 3

[B2] Uthman OA Effect of low birth weight on infant mortality: analysis using Weibull hazard model Int J of Epidemiol 2008 6 1 8

[B3] Daynia EB Tobias FC Peter AC Determinants of survival in very low birth weight neonates in a public sector hospital in Johannesburg BMC Pediatrics 2010 10 30 10 30 20444296 10.1186/1471-2431-10-30 PMC2885379

[B4] Mathew TJ MacDorman MF Infant mortality statistics from the 2003 period linked birth/infant death data set National vital statistics reports 2006 54 16 1 30 16711376

[B5] Kane F Edward MC Gender imbalance in infant mortality: a cross-sectional study of social structure and female infanticide Soc Sci Med 2006 62 360 374 10.1016/j.socscimed.2005.06.006 16046041

[B6] Naeye RL Burt IS Wright DI Neonatal mortality: the male advantage Pediatrics 1971 48 6 902 906 5129451

[B7] Khoury MJ Marks JS McCarthy BJ Factors affecting the sex differential in neonatal mortality Am J Obstet Gynecol 1985 151 6 777 782 3976790 10.1016/0002-9378(85)90518-6

[B8] Plateau State Government: Visit Plateau [Online] 2004 Cited: November 16, 2010.[Available from: http://www.plateaustategov.ng]

[B9] Centres for disease control and prevention: Epi Info 2005 3.3.2 cited May 8 2010.[Available from: http://www.cdc.gov/epiinfo/]

[B10] Clarke M Draper ES James D Mckeeever P Perkins MJ Wood S Confidential enquiry into stillbirths and deaths in infancy (CESDI) 1994-5- one of the Trent infant mortality and morbidity studies 1996 Leicester: Trent institute for health services research

[B11] Ojukwu JU Ogbu CN Analysis and outcome of admissions in the special care baby unit of Ebonyi state university teaching hospital, Abakaliki Journal of Coll of M 2005 9 2 93 96

[B12] Ezeaka VC Ekure EN Iroha EO Egri-Okwaji MT Outcome of low birth weight neonates in a tertiary health care centre in Lagos, Nigeria Afr J Med Sci 2004 33 4 299 303 15977435

[B13] Lams JD Romero R Culhane JF Goldenberg RL Primary, secondary and tertiary interventions to reduce the morbidity and mortality of preterm birth The lancet 2008 371 9607 164 175 10.1016/S0140-6736(08)60108-7 18191687

[B14] Human resources and skills development Canada 2011 [online] cited on October 1st 2011.[ Available:http://www.hrsdc.gc.ca ]

[B15] Herman AA Hastie TJ An analysis of gestational age, neonatal size and neonatal death using nonparametric logistic regression J of Clin Epidemiol 1990 43 11 1179 1190 10.1016/0895-4356(90)90019-L 2243255

[B16] Wilcox AJ Skjaerven Birth weight and perinatal mortality: the effect of gestational age Am J Public Health 1992 82 3 378 382 10.2105/AJPH.82.3.378 1536353 PMC1694365

[B17] Makhoul IR Sujov P Smolkin T Lusky A Reichman B Pathogen-specific early mortality in very low birth weight infants with late -onset sepsis A national surv Clin Infectious Diseases 2005 40 2 218 224 10.1086/426444 15655738

[B18] United Nations Reduce child mortality 2008 21 3 The millennium development goals report New York 340 356

