# Supplementary material for: The effects of birth weight and gender on neonatal mortality in north central Nigeria
Source: BMC Res Notes. 2011 Dec 24;4:562. doi: 10.1186/1756-0500-4-562 (PMC3279327; doi:10.1186/1756-0500-4-562)
Supplement: Additional file 1 — Table S1. Z-test of Gestational Age (GA) and Mortality, Table S2. Neonatal Mortality by Cause of Death. [file 1756-0500-4-562-S1.PDF]

Additional file 1

Table S1: Z-test of Gestational Age (GA) and Mortality

	GA(weeks)	N	Mean	Std.Dev.	z	df	Sig(2-tailed)	Mean difference	95%c.f interval Lower	Upper
**Group Statistics**										
Mortality	**>or=37**	**191**	**0.01**	**0.074**						
	**<37**	**87**	**0.13**	**0.343**						
**Independent samples test**										
Mortality				**0.027**	**4.81**	**277**	**0.000**	**0.129**	**0.181**	**0.076**

Table S2: Neonatal Mortality by Cause of Death

	Total	NNS+ Facial Trauma	NNS	NNJ	NNS +NNJ	Birth asphyxia	Malaria
**PRE-TERMS**							
**Number Admitted**	**87**						
**Mortality**	**11**	**-**	**8**	**-**	**2**	**1**	**-**
**TERMS**							
**Number Admitted**	**191**						
**Mortality**	**1**	**1**	**-**	**-**	**-**	**-**	**-**
